# Involvement of the RND efflux pump transporter SmeH in the acquisition of resistance to ceftazidime in *Stenotrophomonas maltophilia*

**DOI:** 10.1038/s41598-019-41308-9

**Published:** 2019-03-20

**Authors:** Paula Blanco, Fernando Corona, José Luis Martínez

**Affiliations:** Centro Nacional de Biotecnología, CSIC, 28049 Madrid, Spain

## Abstract

The emergence of antibiotic resistant Gram-negative bacteria has become a serious global health issue. In this study, we have employed the intrinsically resistant opportunistic pathogen *Stenotrophomonas maltophilia* as a model to study the mechanisms involved in the acquisition of mutation-driven resistance to antibiotics. To this aim, laboratory experimental evolution studies, followed by whole-genome sequencing, were performed in the presence of the third-generation cephalosporin ceftazidime. Using this approach, we determined that exposure to increasing concentrations of ceftazidime selects high-level resistance in *S. maltophilia* through a novel mechanism: amino acid substitutions in SmeH, the transporter protein of the SmeGH RND efflux pump. The recreation of these mutants in a wild-type background demonstrated that, in addition to ceftazidime, the existence of these substitutions provides bacteria with cross-resistance to other beta-lactam drugs. This acquired resistance does not impose relevant fitness costs when bacteria grow in the absence of antibiotics. Structural prediction of both amino acid residues points that the observed resistance phenotype could be driven by changes in substrate access and recognition.

## Introduction

The evolution and dissemination of antibiotic resistance has become one of the major threats for public health worldwide, being the spread of multidrug resistant (MDR) Gram-negative bacteria one of the main problems nowadays^1^. *Stenotrophomonas maltophilia* is an opportunistic nosocomial pathogen responsible for causing a variety of infections, with high morbidity and mortality especially in patients with underlying pathologies, as cystic fibrosis, and in those who are immunocompromised^2–4^. One of the main characteristics of this bacterium is its low susceptibility to a broad range of antibiotics, which entails a difficulty in the treatment of the infections that it causes^5,6^.

Among the elements that determine the intrinsic low susceptibility of *S. maltophilia* to antibiotics, it is important to highlight the low permeability of its membrane, as well as the presence in its genome of a number of intrinsic resistance genes that encode antibiotic-modifying enzymes, the quinolone resistance protein SmQnr and MDR efflux pumps, being of higher relevance those belonging to the resistance nodulation division (RND) family^7–11^. Few therapeutic options are currently in use for treating *S. maltohilia* infections. Among them, combinations of antibiotics including classical ones, as trimethoprim/sulfamethoxazole, are in use^12–15^. Despite *S. maltophilia* contains in its genome two intrinsic beta-lactamases, dubbed L1^9^ and L2^10^, they are not particularly active against ceftazidime at the level they are expressed in several clinical *S. maltophilia* isolates^16–18^. Consequently, use of ceftazidime, mainly in combination with other antibiotics, has been suggested for treating *S. maltophilia* infections^19–23^, although the success of these treatments might be challenged given the large number of antibiotic resistance genes that *S. maltophilia* harbours. Indeed, it has been shown that mutants overexpressing L1 and L2 beta-lactamases and presenting a reduced susceptibility to ceftazidime can be easily selected *in vitro*^17,24^. Further, recent work has shown that *S. maltophilia* beta-lactamase-overexpressing ceftazidime-resistant mutants lacking a functional Mpl are frequently selected in clinics^25^. Despite these findings, little is still known about other potential resistance mechanisms, besides beta-lactamase overexpression, that might impact not only *S. maltophilia* susceptibility to ceftazidime, but to other antibiotics as well. To analyze this possibility, we challenged *S. maltophilia* with increasing concentrations of ceftazidime and analyzed the mutations that arised after this selection. Notably, we found that one of the first mutations to be selected was located in *smeH*, which encodes the transporter protein of the SmeGH RND efflux pump. RND efflux pumps are involved in the intrinsic resistance to several antibiotics when expressed at a basal level and their induction by environmental signals or conditions sensed by bacteria can lead to transient antibiotic resistance^26,27^. In addition, mutants overexpressing these efflux pumps are selected by antibiotics *in vitro* and are regularly isolated from infected patients^28–34^.

Although some few publications address that mutations in the structural elements of efflux pumps can alter their specificity and hence contribute to antibiotic resistance^35–40^, most works analyzing the role of efflux pumps in acquired antibiotic resistance focus on their level of expression^41–50^, and not on the allelic variations in the genes encoding the efflux pump. Herein, we show that changes in the structure of SmeH, without the need of its overexpression, reduce the susceptibility of *S. maltophilia* to different antibiotics, including ceftazidime.

## Results

### Experimental evolution upon ceftazidime challenge leads to high levels of resistance in *S. maltophilia* populations

**Figure 1:**
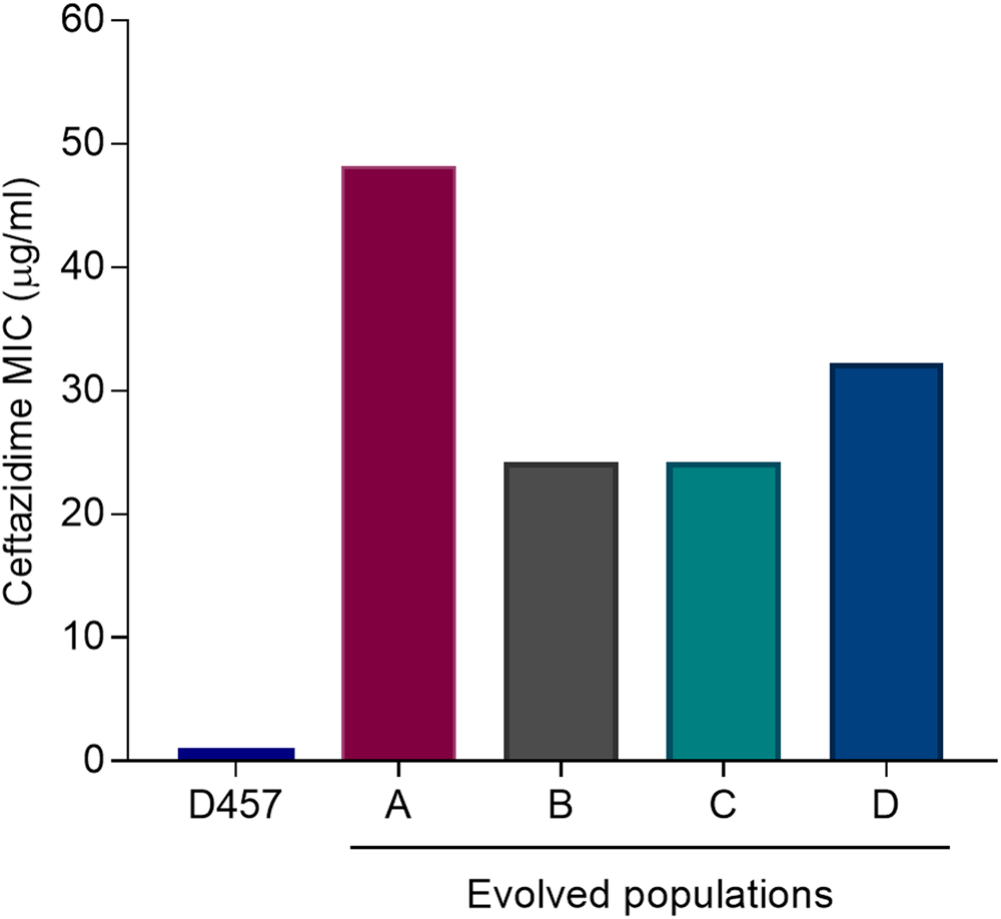
MICs for ceftazidime of the four *S. maltophilia* evolved populations. MIC was determined after 30 days of experimental evolution in the presence of increasing concentrations of ceftazidime in the populations A, B, C and D. The parental strain D457 was used as a reference. MIC, minimum inhibitory concentration.

In order to elucidate how ceftazidime challenge impacts the acquisition of ceftazidime resistance, four independent *S. maltophilia* D457 cultures were serially passaged during 30 days of evolution in the presence of increasing concentrations of ceftazidime, reaching 32-fold the starting concentration at the end of the experiment. Four control cultures were also performed in the absence of antibiotic to track the selection of medium-adaptive mutations, not involved in resistance to ceftazidime challenge. Prior to the final evaluation of the evolved populations’ susceptibility to ceftazidime, serial daily passages in LB medium without ceftazidime were performed for 3 days in order to exclude a possible induction of resistance caused by the previous ceftazidime exposure. Ceftazidime susceptibility was then evaluated in the four evolved populations (A, B, C and D) by Etest. As shown in Fig. 1, all the populations reached high levels of ceftazidime resistance after 30 days of evolution in comparison with the parental strain D457.

### Selected mutations and temporal dynamics of *S. maltophilia* evolution in the presence of ceftazidime

With the aim of assessing the genetic mechanisms involved in ceftazidime resistance, the genomic DNAs of the evolved populations in presence of ceftazidime and the controls, grown in absence of antibiotics as well as of the parental strain, were extracted and sequenced. The coverage of the sequence was 106 on average, ranging from 90 to 113. The presence of mutations was determined in all populations as described in Methods, and confirmed by PCR amplification and Sanger sequencing. Only those mutations that were selected upon antibiotic selective pressure, but were absent in the populations evolved in the absence of selection, were taken into consideration. Table 1 shows those genetic changes that were identified after the whole genome sequencing (WGS) data analysis, and subsequently confirmed by Sanger sequencing, present in the ceftazidime-treated populations and absent in controls. In total, changes were found in 11 different genes. Most mutations were single nucleotide polymorphisms (SNPs) leading to amino acid substitutions. Among them, mutations in *smeH* were found in the four evolved populations, whereas mutations in *phoQ* were selected in three of them. Mutations in the penicillin binding protein (PBP) *ftsI* in population A, or in genes encoding for hypothetical proteins, as SMD_2719 also in population A, and SMD_0260 in population B, were also selected. Insertions or deletions leading to frame shifts were also found in other genes. For instance, a 188-bp deletion in the lipopolysaccharide biosynthesis regulator *yciM* was detected in population C, and a 10-bp deletion in the permease component *yrbE* of an uncharacterized ABC transporter was selected in population D. In addition, an insertion sequence (IS) belonging to the IS5 family was also involved in the disruption of the gene *smd_0534*, which encodes a hypothetical protein, in population A. Noteworthy, and although every independent population seems to have a different resistance pattern, it was possible to find in the four antibiotic-evolved populations the same amino acid substitution (P326Q) in SmeH, the transporter protein of the SmeGH RND efflux pump.

**Table 1:** WGS-identified mutations in ceftazidime-evolved populations.

Population	Gene	Localization	Type	Nucleotide change	Amino acid change	Frequency (%)	Detected day of emergence
A	*smeH*	3061160	SNV	C → A	P326Q	98.7	5
	*SMD_2719*	3026930	SNV	A → C	V232G	51.6	20
	*phoQ*	315221	SNV	T → A	I76N	100	25
	*ftsI*	736289	SNV	C → A	A592D	100	25
	*SMD_0534*	613290	Ins	Ins of 1.037 bp	V398fs	—	30
B	*smeH*	3061160	SNV	C → A	P326Q	100	5
	*smeH*	3062171	SNV	A → G	Q663R	100	20
	*SMD_0260*	314100	SNV	A → G	K88R	70.1	25
C	*smeH*	3061160	SNV	C → A	P326Q	100	5
	*yrbC*	4744239	SNV	C → T	Q126*	32.3	30
	*yciM*	2035186	Del	Del of 188 bp	A206fs	—	30
	*phoQ*	315914	SNV	C → T	S307L	41.2	30
	*phoQ*	315965	SNV	C → T	P324L	37.5	30
D	*smeH*	3061160	SNV	C → A	P326Q	100	5
	*smeH*	3062171	SNV	A → G	Q663R	100	20
	*phoQ*	315236	SNV	C → T	P81L	99.2	20
	*yrbE*	4742678	Del	ATCGCCGTCG → —	I49fs	65.3	30
	*SMD_1278*	14224481	Ins	— →TGACTT	F91_G92insDF	57.1	30
	*mrkC*	685444	Del	GGCTTC → —	G187_F188del	76.3	30

SNV: single nucleotide variant; Ins: insertion; Del: deletion; Frequency (%): percentage of reads that contain the variation within a heterogeneous population; * STOP codon.

The temporal dynamics of the different genetic changes were determined by amplifying and sequencing each of the mutated genes from stored samples of the four populations at days 5, 10, 15, 20, 25 and 30 (Table 1). The most remarkable aspect from these dynamics is the detection of the amino acid substitution P326Q in SmeH at day 5 of evolution in the four treated populations, when the ceftazidime concentration was 1 µg/ml. Further, in populations B and C, the following substitution in emerging was a second amino acid change in SmeH, Q663R, which was detected at day 20 when the ceftazidime concentration was 8 µg/ml. The remaining acquired genetic changes were found between days 20 and 30 of evolution.

### Effect of SmeH substitutions on resistance and fitness

Since *smeH* mutations were found in the four ceftazidime-exposed populations and there are no previous studies regarding the contribution of the SmeGH efflux pump to antibiotic resistance, we decided to determine the role of both P326Q and Q663R substitutions in *S. maltophilia* ceftazidime resistance. In order to do this, both mutations were recreated in the parental strain D457, either together or separately, obtaining the strains PBT101 (P326Q), PBT102 (Q663R) and PBT103 (P326Q; Q663R). The MIC of ceftazidime was determined for the three recreated strains, as well as for the parental strain D457. While P326Q substitution was able to increase more than 5-fold the ceftazidime MIC (4 µg/ml), the second substitution Q663R did not lead to a change in the susceptibility by itself (0.75 µg/ml). Nevertheless, the presence of both mutations led to a 10-fold change in the MIC levels (8 µg/ml).

**Figure 2:**
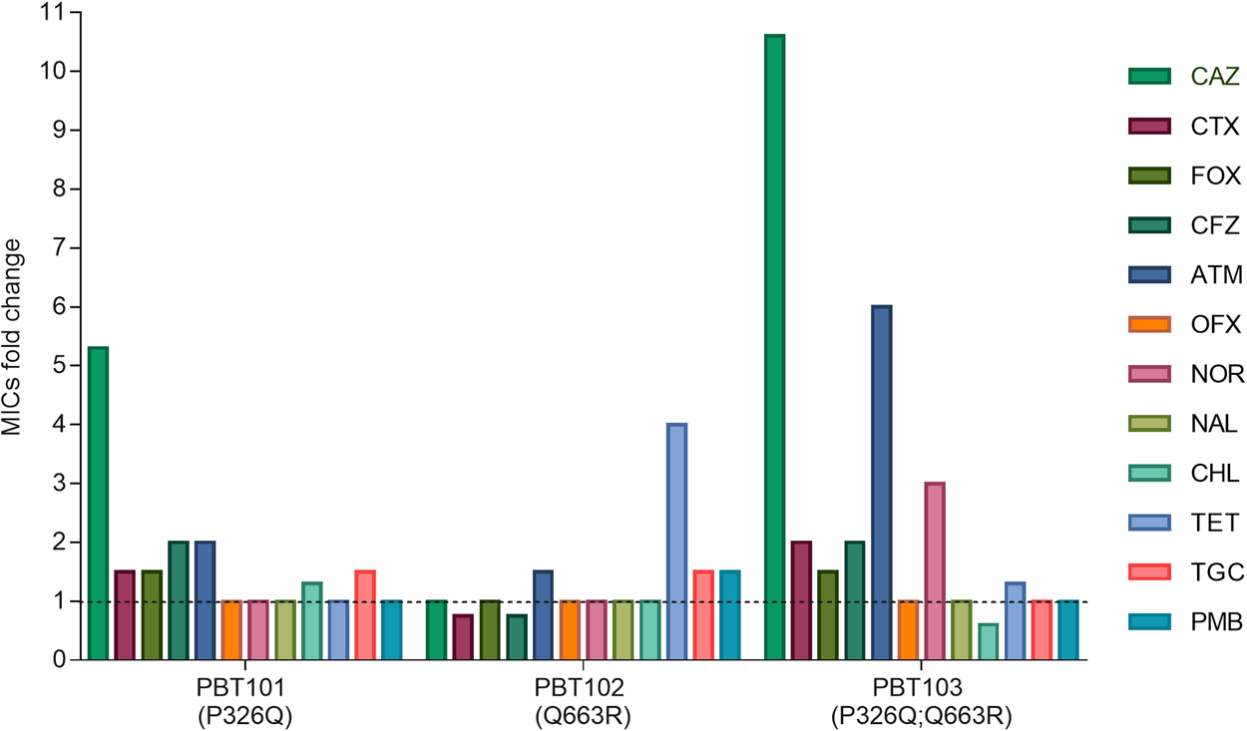
Susceptibility of the *S. maltophilia* mutants to different antibiotics. MICs were measured in the three recreated mutants PBT101 (P326Q), PBT102 (Q663R) and PBT103 (P326Q; Q663R). Fold changes were determined using the MIC values of the wild-type strain D457 as reference (dotted line). MIC, minimum inhibitory concentration; CAZ, ceftazidime; CTX, cefotaxime; FOX, cefoxitin; CFZ, cefazolin; ATM, aztreonam; OFX, ofloxacin; NOR, norfloxacin; NAL, nalidixic acid; CHL, chloramphenicol; TET, tetracycline; TGC, tigecycline; PMB, polymyxin B.

To explore whether the ceftazidime resistance mutations in *smeH* lead to cross-resistance and/or collateral sensitivity to other antibiotics, changes in the susceptibility to several classes of antibiotics were measured in the strains PBT101, PBT102 and PBT103 using the parental strain D457 as a reference. The MICs of the different antibiotics are shown in Supplementary Table S1. As observed in Fig. 2, a two-fold increase in the MIC was obtained for the beta-lactams cefazolin and aztreonam in the PBT101 strain. In the case of the PBT103 strain, the fold change for these antibiotics was 2-fold and 6-fold, respectively. Further, cross-resistance in this strain was also observed for cefotaxime (2-fold change) and the quinolone norfloxacin (3-fold change). As happened with ceftazidime, no changes in the MICs for beta-lactams were observed in the PBT102 strain; however, a 4-fold change in the MIC was obtained for tetracycline. No collateral sensitivity was observed for any of the tested antibiotics.

**Figure 3:**
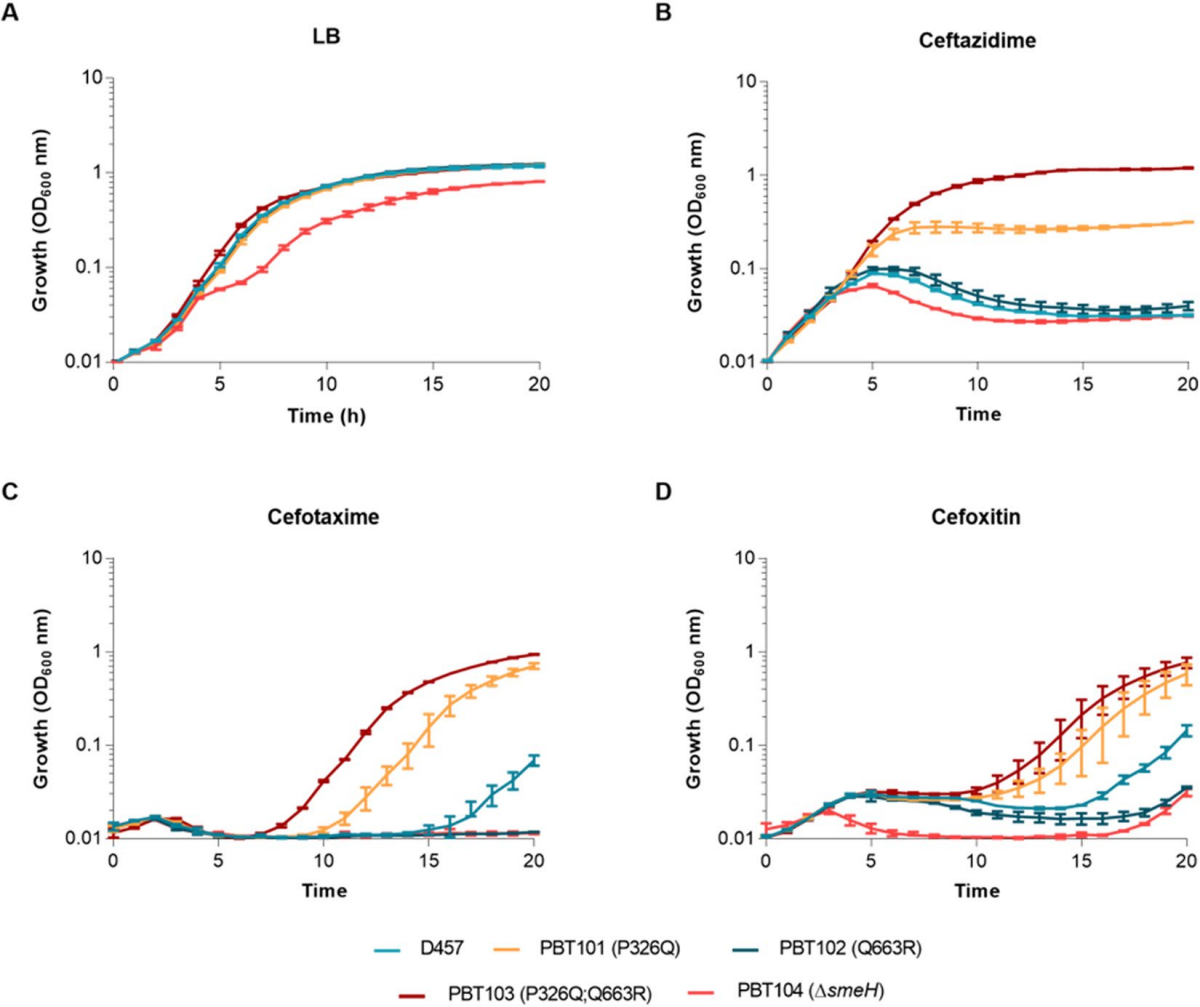
Effect of beta-lactams on the growth of *S. maltophilia* strains. Growth curves were performed for the three *S. maltophilia* recreated mutants PBT101 (P326Q), PBT102 (Q663R) and PBT103 (P326Q; Q663R), the *smeH*-defective strain PBT104 and the parental strain D457 in the absence (**A**) or presence of ceftazidime (4 µg/ml) (**B**), cefotaxime (256 µg/ml) (**C**) and cefoxitin (128 µg/ml) (**D**) during 20 h. Error bars show standard deviations from three independent experiments.

In order to study in more detail the observed resistance to beta-lactams in the PBT101 and PBT103 strains, a more sensitive method based on bacterial growth was applied. The OD_600_ was recorded in the presence of ceftazidime, cefotaxime and cefoxitin for 20 h. Strains D457, PBT102 and PBT104 were also included in the experiment. Data shown in Fig. 3B, C and D, proved that only mutants PBT101 and PBT103 were able to grow in the presence of the three selected antibiotics, being the resistance of the PBT103 strain higher than that of PBT101. Meanwhile, the PBT102 mutant displayed a growth similar to the observed for the wild-type strain in all conditions, confirming the above-obtained MIC results. We also evaluated the effect of these mutations on fitness cost. As shown in Fig. 3A, the strains PBT101, PBT102 and PBT103 had no growth defect in comparison with the parental strain in LB medium; only the PBT104 strain, which lacks *smeH*, showed a slight growth impairment. These results point that fitness is not compromised in an antibiotic-free medium when *smeH* mutations are present.

### Role of SmeGH efflux pump in the intrinsic resistance and the physiology of *S. maltophilia*

**Figure 4:**
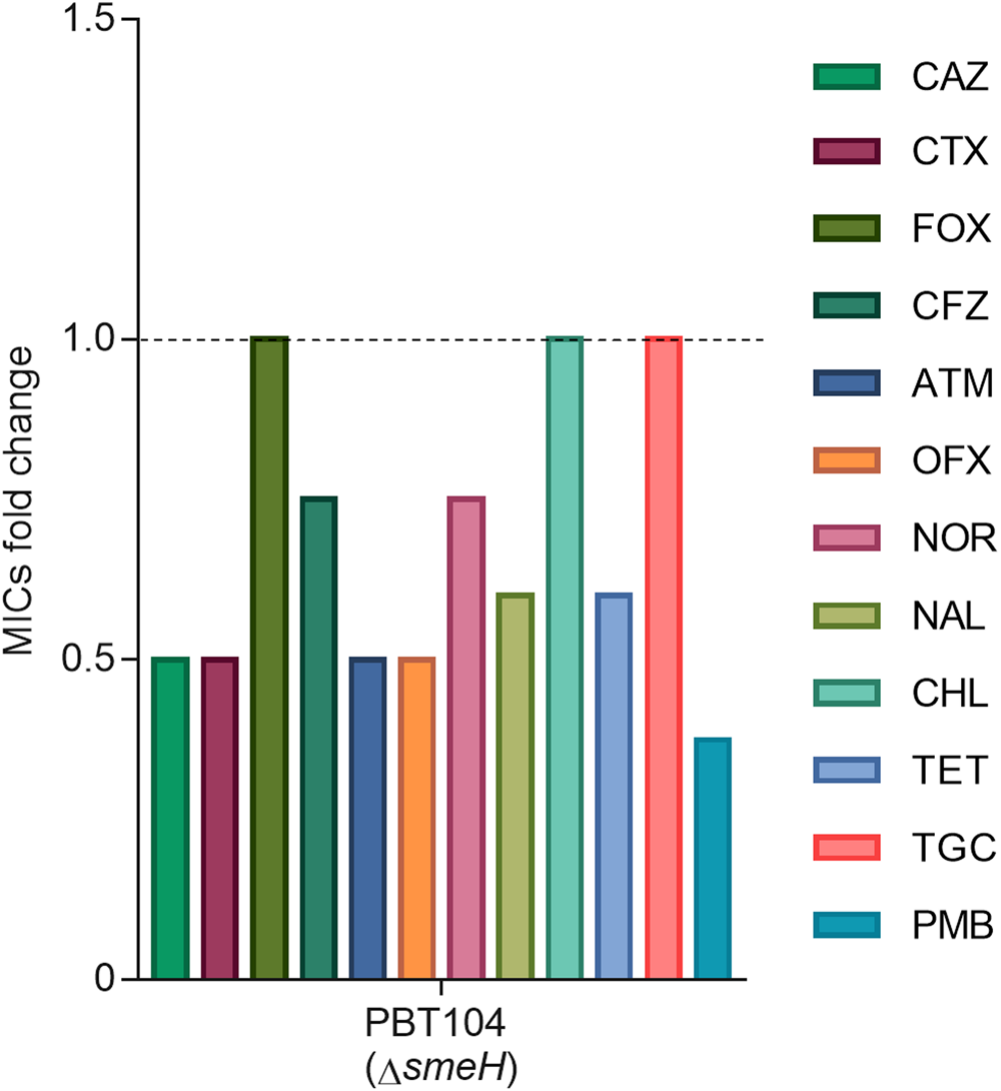
Susceptibility of the *S. maltophilia smeH*-defective mutant to different antibiotics. MICs were measured in the PBT104 strain (*ΔsmeH*) and the fold changes were determined using the MIC values of the wild-type strain D457 as reference (dotted line). MIC, minimum inhibitory concentration; CAZ, ceftazidime; CTX, cefotaxime; FOX, cefoxitin; CFZ, cefazolin; ATM, aztreonam; OFX, ofloxacin; NOR, norfloxacin; NAL, nalidixic acid; CHL, chloramphenicol; TET, tetracycline; TGC, tigecycline; PMB, polymyxin B.

Besides studying its contribution to the acquisition of resistance, we decided to assess the role of the SmeGH efflux pump in the *S. maltophilia* intrinsic resistance through the generation of a *smeH*-deficient mutant. In addition, we wanted to address whether the original substrates of the wild-type allele of the efflux pump are the same as those of the selected mutants and hence the amino acid substitutions just modify the efficiency; or they are different and the mutations alter the substrates specificity of SmeH. The MICs for ceftazidime, as well as for the other antibiotics, were determined in the PBT104 (*ΔsmeH*) strain and are shown in Supplementary Table S1. As shown in Fig. 4, the *smeH*-deficient mutant PBT104 showed an increased susceptibility to ceftazidime and to almost all tested antibiotics, excepting chloramphenicol and tigecycline, in comparison with the wild-type strain. In the case of cefoxitin, no changes in the MIC were observed; however, as shown in Fig. 3D, the PBT104 mutant presents a more impaired growth, as compared to the wild-type strain, in the presence of this beta-lactam. These results suggest that these antibiotics could be substrates of the SmeGH efflux pump, which would then be involved in *S. maltophilia* intrinsic resistance.

In addition to commonly-used antibiotics, we evaluated the implication of the SmeGH efflux pump in the susceptibility to other antimicrobial toxic compounds. As shown in Table 2, the *smeH*-deficient strain PBT104 was more susceptible than the parental strain to the oxidative stress-generating compounds menadione and tert-butyl hydroperoxide, as well as to the biocide benzalkonium chloride and to the plant-derived compound naringenin. Changes in the MICs were also observed for some of the compounds in the *smeH*-mutant alleles selected in presence of ceftazidime. A two-fold increase in the MIC was observed in the PBT101 (P326Q) strain for all the tested biocides (hexachlorophene, benzalkonium chloride and triclosan). Further, a higher susceptibility to menadione was detected in the PBT102 (Q663R) and PBT103 (P326Q; Q663R) strains, and a lower MIC was also obtained for tert-butyl hydroperoxide in the PBT103 strain. These data suggest that, besides its contribution to antibiotics resistance, SmeGH might be involved in the detoxification of some other toxic compounds and the amino acid substitutions in SmeH selected in presence of ceftazidime could have different effects on the capability of this efflux pump for extruding other compounds.

**Table 2:** MICs of antimicrobial compounds for *S. maltophilia* strains.

Strain	MD (mM)	TBHP (mM)	NGEN (mM)	HCP (μg/ml)	BAC (μg/ml)	TRI (μg/ml)
D457	1.25	0.62	25	2	6.25	25
PBT101 (P326Q)	1.25	0.62	25	4	12.5	50
PBT102 (Q663R)	0.62	0.62	25	2	6.25	25
PBT103 (P326Q; Q663R)	0.50	0.31	25	2	6.25	25
PBT104 (*ΔsmeH*)	0.12	0.31	3.12	2	1.56	25

MIC, minimum inhibitory concentration; MD, menadione; TBHP, tert-butyl hydroperoxide; NGEN, naringenin; HCP, hexachlorophene; BAC, benzalkonium chloride; TRI, triclosan.

**Figure 5:**
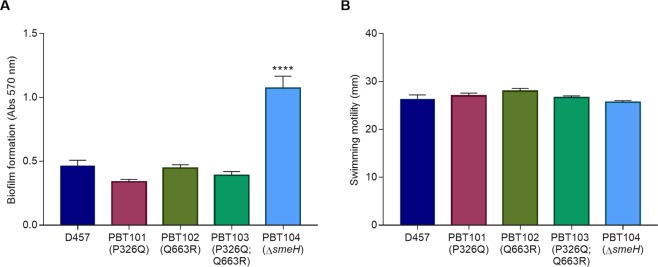
Biofilm formation and swimming motility of *S. maltophilia* strains. (**A**) The biofilm formation assay was carried out by measuring the absorbance at 570 nm of crystal violet after 48 h incubation. Error bars show standard deviations from eight replicate samples. ****Indicates *P* < 0.001 calculated by one-way ANOVA test. (**B**) The swimming motility assay was performed by measuring the growth area after 48 h incubation on LB semisolid agar (0.3%) plates. Error bars show standard deviations from three independent experiments.

The role of SmeGH in *S. maltophilia* physiology was also studied through the evaluation of virulence-related characteristics, as biofilm formation and swimming motility. As shown in Fig. 5A, the PBT104 (*ΔsmeH*) strain presented an enhanced ability to form biofilm in comparison with the parental strain D457, while no significant differences in the swimming ability were detected among the different strains (Fig. 5B).

### Ceftazidime-selected SmeH substitutions are likely located in the vicinity of the access and deep binding pockets of the transporter protein

**Figure 6:**
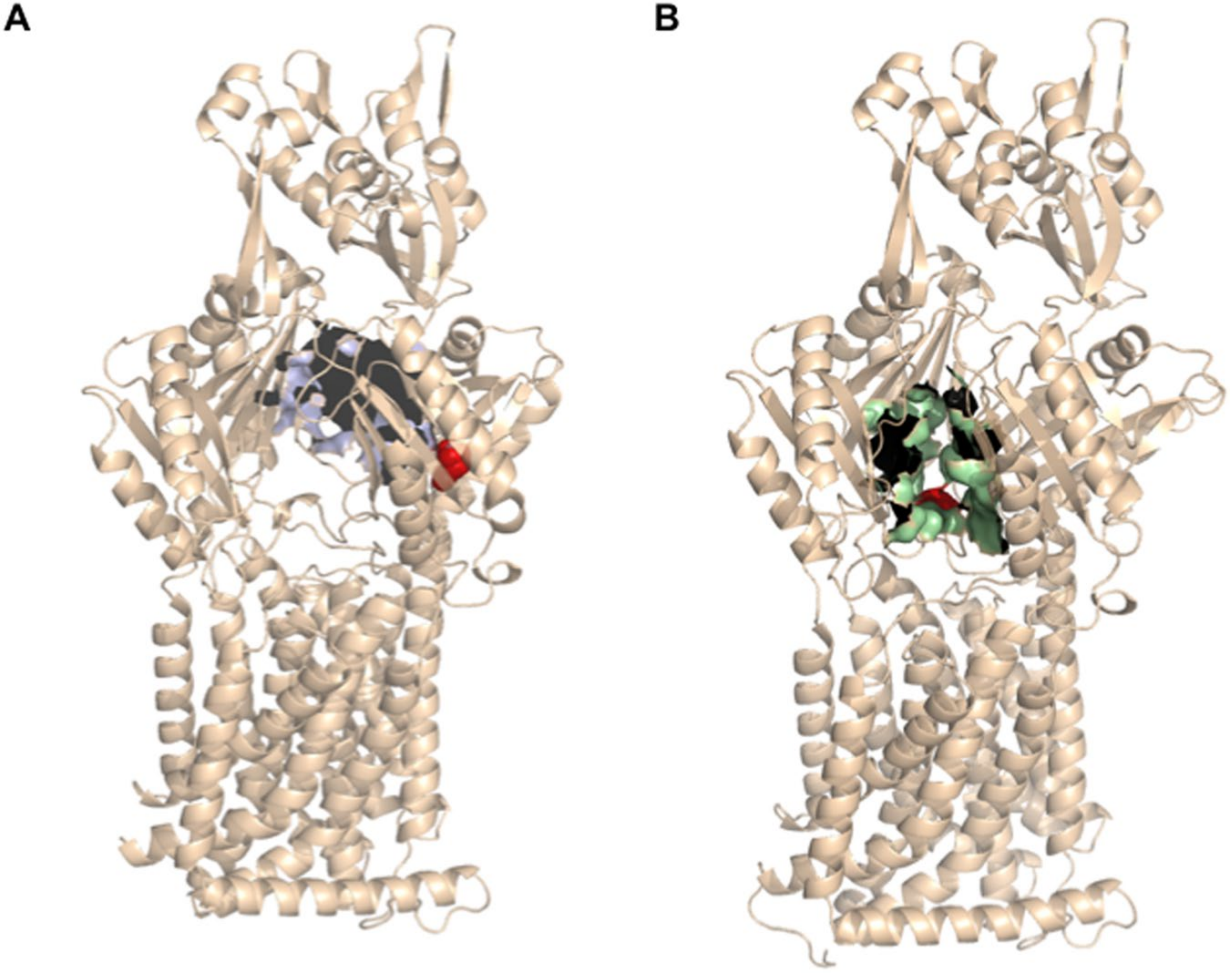
Mapping of SmeH-amino acid residues on the 3D structure of AcrB. (**A**) Surface representation of the predicted deep binding pocket (violet) on the AcrB tight monomer (PDB code 4DX7.B). P326 is represented by red spheres. (**B**) Surface representation of the predicted access pocket (green) on the AcrB loose monomer (PDB code 4DX7.A). V672, the Q663-corresponding residue in AcrB, is represented by red spheres.

With the purpose of elucidating the molecular basis behind the phenotype conferred by both SmeH mutations, a structural alignment of the *S. maltophilia* SmeH transporter was performed with the *Escherichia coli* AcrB efflux protein sequence, showing that both proteins are homologous (Template modeling score 0.97, sequence identity 0.51, coverage 0.98). The alignment showed that residue P326 is conserved in both transporter proteins (Supplementary Fig. S1); consequently, the available crystal structure of AcrB (PDB ID code 4DX7.B^37^) was used for predicting the effect of the mutation P326Q in *S. maltophilia*. It has been described that AcrB monomers can adopt one of three conformations, labeled as loose (L), tight (T) and open (O), depending on the step of the drug export process (access, binding, and extrusion, respectively)^51^. Using these templates, the P326 residue was predicted to be located in the proximities of the deep binding pocket in the tight (T), or binding, monomer of AcrB (Fig. 6A). Q663, which corresponds to the V672 in AcrB, was also represented in the AcrB crystal structure (PDB ID code 4DX7.A^37^), and it was mapped at the bottom of the access pocket of the efflux protein (Fig. 6B).

**Figure 7:**
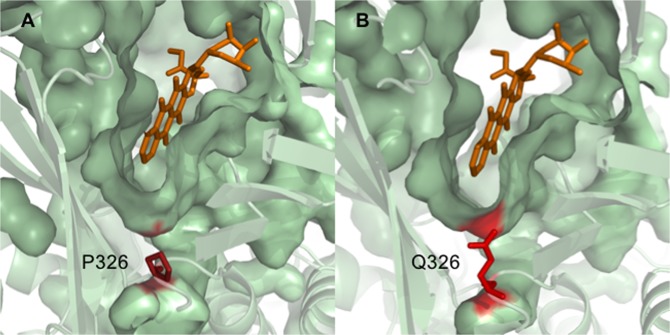
Effect of P326Q substitution on the residue orientation in the SmeH deep binding pocket. Surface representation of the deep binding pocket on the AcrB tight monomer (PDB code 4DX7.B) with the wild-type residue P326 (**A**) and the mutant allele Q326 (**B**). Both residues are represented by red sticks and doxorubicin is represented in orange.

Since P326 is conserved in both species, we wanted to study the effect of the P326Q substitution in the AcrB crystal in its doxorubicin-bound form (PDB ID code 4DX7.B^37^) in order to have a reference of the deep binding pocket conformation when a known substrate is bound inside. In its wild-type form, the protamine residue is mapped under the deep binding pocket of the transporter protein, but contactless (Fig. 7A). However, when Q326 is represented, the glutamine residue can be found pointing into the deep binding pocket cavity (Fig. 7B). Although in this simulation the glutamine residue does not seem to interact with the cocrystallized substrate directly, it might establish contact with other residues that are part of the binding pocket and change in some way the affinity for the antibiotic.

## Discussion

Experimental evolution studies provide important information on the genetic adaptive changes underlying the acquisition of different phenotypes by bacterial populations^52^, including antibiotic resistance^53^. In the current work, we have used such approach for analyzing the evolutionary trajectories of the relevant nosocomial pathogen *S. maltophilia* towards ceftazidime resistance. As it happens in the case of other studies on evolution towards antibiotic resistance^54^, the evolutionary trajectories followed by *S. maltophilia* parallel cultures submitted to antibiotic selective pressure, shared some common elements, namely mutations in the efflux pump transporter gene *smeH* (present in the four evolved populations) and in the two-component sensor histidine kinase *phoQ* (three out of the four evolved populations), indicating that, as stated in^54,55^, evolution towards antibiotic resistance might present some degree of predictability. Some genes that present mutations in the evolved bacterial populations have been previously described to have a role in antimicrobial resistance in other microorganisms. This is the case of *ftsI*, a gene encoding a peptidoglycan transpeptidase^56^, whose mutation has been reported to be involved in resistance to beta-lactam drugs^55,57,58^, or *phoQ*, which is involved in polymyxin and antimicrobial peptides resistance^59,60^, although its potential role in beta-lactams resistance has not been studied in detail. The other genes presenting mutations involved in the acquisition of ceftazidime resistance in *S. maltophilia* have not been described previously to be involved in antibiotic resistance. Namely, mutations in genes encoding the auxiliary (*yrbC*) and the permease components (*yrbE*) of an uncharacterized ABC transporter, or the RND transporter *smeH*, as well as four hypothetical proteins were selected upon antibiotic selective pressure. Temporal analysis of the dynamics of evolution showed that several of the mutations emerged from day 20 of evolution; however, for all the populations, the first step leading to acquisition of resistance to ceftazidime was the P326Q substitution in SmeH, being the first and only mutation detected at day 5 of evolution. A second substitution in SmeH, Q663R, emerged in two of the treated populations days later after P326Q.

Considering the important role that the SmeGH efflux pump seems to have in the acquisition of ceftazidime resistance, we focused on these amino acid substitutions to get more insights into their contribution to resistance. Data showed that strains PBT101 (P326Q) and PBT103 (P326Q; Q663R) exhibited an increase of 5- and 10-fold in the ceftazidime MIC, respectively, while PBT102 (Q663R) remained at the same level of the wild-type strain, indicating this mutation to be neutral concerning ceftazidime resistance in the wild-type genomic background. Meanwhile, the presence of both mutations in the *smeH* gene has a positive effect regarding ceftazidime resistance, having the second mutation an impact just in combination with the first one. In agreement with data derived from the study of the evolution of extended spectrum beta-lactamases^61^, this result supports the notion that epistatic interactions (including neutral mutations) modulate the evolution towards resistance and, consequently, the order of acquisition of mutations is highly relevant for the final phenotypic outcome.

Several RND efflux pumps extrude a broad range of unrelated compounds from the bacterial cell^62^. With the aim of determining whether the P326Q and Q663R changes are specific for ceftazidime resistance or they change *S. maltophilia* susceptibility to other antibiotics, MICs of other drugs were measured. Our results showed that PBT101 exhibited increased resistance to the beta-lactams aztreonam and cefazolin, and PBT103, besides these ones, presented also increased resistance to cefotaxime and the quinolone norfloxacin. Besides, growth-based experiments allowed us to detect an increased resistance against cefotaxime in the PBT101 strain, and against cefoxitin in both PBT101 and PBT103 strains. These data indicate that P326Q and the combination of both P326Q/Q663R in SmeH do not confer specific resistance to ceftazidime, but also to other antibiotics, mostly beta-lactams. Conversely, PBT102 did not show any change in the susceptibility to beta-lactams but it did for tetracycline, suggesting that, while the Q663R substitution does not play any role in beta-lactams resistance by itself, it could lead to tetracycline resistance, a feature that may allow to select this mutation in a wild-type background, independently of the P326Q mutation, under tetracycline challenge.

Finally, the hyper-susceptibility of the *smeH*-defective mutant PBT104 to some beta-lactam drugs, as well as to quinolones, polymyxin B and tetracycline, suggests that SmeGH is an intrinsic resistant determinant for these antibiotics. In addition, the fact that this mutant presents changes in the susceptibility to the same beta-lactams as PBT101 and PBT103 does indicate that the mutations selected in *smeH* along experimental evolution likely alter the efficiency of extrusion of these substrates.

According to our results, SmeGH might not only be involved in resistance to antibiotics, but also to other toxic compounds belonging to different families, as the oxidative stress agents menadione and tert-butyl hydroperoxide, the plant-derived compound naringenin, and the biocide benzalkonium chloride. Other *S. maltophilia* RND efflux pumps have been previously reported to be involved in the extrusion of toxic molecules. For instance, SmeDEF, besides antibiotics, is able to extrude triclosan^63^ and the plant exudate phloretin^64^. Since RND efflux pumps are important bacteria detoxification elements^65^, it is possible that some of these compounds are substrates of SmeGH, which would participate in their detoxification. Further, as happened with beta-lactams, PBT101, PBT102 and PBT103 strains also showed changes in their MICs to some of these toxic compounds, indicating that the amino acid substitutions present in SmeH in these mutants and selected in presence of ceftazidime could interfere in the capability of this efflux pump for extruding these molecules.

In order to get more insights into the physiological roles that the SmeGH efflux pump could have, biofilm formation and swimming motility assays were assessed in all the strains. While the swimming ability was neither compromised nor increased in any of the *S. maltophilia* strains, deletion of the transporter component SmeH led to an enhanced biofilm production in the PBT104 strain. Although the molecular mechanisms regarding this phenotype remain to be clarified, it is possible that the SmeGH efflux pump contributes negatively to biofilm formation by the regulation of biofilm-related genes, as happens with the ABC transporter lm.G_1771 in *Listeria monocytogenes*^66^, or by the accumulation of toxic molecules that could trigger the expression of genes involved in biofilm formation through a general stress response, as proposed by Bazzini *et al*. in the case of *Burkholderia cenocepacia*^67^.

It has been generally assumed that, in the absence of selection, the acquisition of antibiotic resistance will impose a burden to the resistant organism, which would be outcompeted by the susceptible ones^68–70^. For instance, mutations leading to the overproduction of MDR efflux pumps can lead to the extrusion of beneficial compounds outside the cell, as well as a non-physiological expense of energy, having an impact on the bacterial fitness unless compensation mechanisms are triggered^71^ or secondary compensatory mutations are selected^72^. With the aim of determining the effect on the *S. maltophilia* fitness of each of the *smeH* mutations alone and in combination, all the strains were grown in absence of antibiotics and their growth was recorded. The only strain impaired in growth was the *smeH*-deficient PBT104, while none of the resistant mutants showed any deficiency regarding bacterial growth, indicating that: I) the removal/inhibition of intrinsic resistance determinants may impair bacterial fitness. In other words, intrinsic resistance determinants might be under positive selection pressure; II) the presence of the analyzed antibiotic resistance mutations, separate or together, does not lead to a decrease on the *S. maltophilia* fitness in an antibiotic-free environment; and III) the second mutation in *smeH* does not seem to have been selected for compensating the potential fitness cost of the first mutation, at least in the recreated strains background. Previous studies have shown that some resistant mutations are cost-free^73–75^, or even confer an enhanced fitness and virulence in infected hosts^76,77^. This fact makes that bacteria harbouring no-cost resistance mutations, as those described in the current article, to be more likely to persist in the absence of antibiotics^78^.

Acquisition of resistance in *S. maltophilia* regarding RND efflux pumps, is usually associated with mutations in their regulatory genes^28–30^. Nevertheless, this is the first report of mutations occurring in an efflux pump transporter protein that lead to a change in the susceptibility to several antibiotics belonging to different categories in this bacterium. Previous studies have shown that amino acid substitutions in the transporter proteins of RND efflux pumps belonging to other bacterial species can change the susceptibility to some antibiotics^35–40^. In these reports, authors highlight the importance of those substitutions located inside or near the transporter binding pocket, where they are of relevance for the recognition and/or accommodation of certain substrates. To better understand how the amino acid substitutions in SmeH impact in the observed acquisition of resistance in *S. maltophilia*, we mapped both residues P326 and Q663 using the AcrB structure from *E. coli* as a model, since no crystal structures for SmeH or other *S. maltophilia* transporters are available. The location of both residues suggests that they could be involved in the entrance and/or recognition of the SmeH substrates, which would have an effect in the resistance levels to these compounds. Since the P326 residue is conserved in both proteins, we performed an *in silico* mutagenesis on the AcrB structure in its doxorubicin-bound form in order to determine whether the mutation P326Q had an impact in the deep binding pocket conformation. According to our prediction, this amino acid change made the Q326 residue to be pointing to the base of the deep binding pocket. In addition to AcrB, P326 is also found to be conserved in the AcrB-homolog MexB from *P. aeruginosa* (Supplementary Fig. S1), as well as in another RND transporter protein from the same bacterium, MexD, corresponding in this case with the P328 moiety. Mao *et al*.^79^ reported that, together with other amino acid substitutions, P328L in MexD increased resistance in *P. aeruginosa* against aztreonam and carbenicillin. This residue was mapped to the large periplasmic loop (LPL) of the RND efflux pump, which can potentially surround the drug-binding pocket. In agreement with this, LPLs have also been reported to play an important role in substrate recognition in AcrB and AcrD from *E. coli*^80^. Although it is unclear how this mutation affects the efflux pump function, the change in the residue orientation might cause interactions with other neighboring residues that are part of the cavity and influence somehow the recognition of the efflux pumps substrates, as beta-lactams, leading to the observed resistance phenotype.

But, why does Q663R only confer changes in beta-lactams susceptibility in combination with P326Q, and not by itself? It has been recently reported that V672, the Q663-equivalent residue in AcrB, might form part of the meropenem binding site of this protein^81^. Besides, our mapping prediction showed that this residue was located at the bottom of the AcrB access pocket. We postulate that changes just in the access cavity could not be enough to have a direct effect on substrate extrusion; however, when P326Q is present and, presumably, binding is ameliorated, the existence of Q663R suppose a beneficial outcome, since both access and recognition of the antibiotic would be improved. Further studies on the structures of RND efflux pump transporters might shed light on this issue.

## Concluding Remarks

Experimental evolution and whole-genome sequencing after ceftazidime exposure, have revealed new mechanisms of acquired resistance in the opportunistic pathogen *S. maltophilia*. Among them, we highlight the selection, in all the ceftazidime-challenged strains, of mutations in the RND efflux pump transporter *smeH* for the first time. Some few publications have reported that mutations in efflux transporters may decrease the susceptibility to antibiotics of bacterial pathogens^35–40^. However, this is the first formal demonstration showing that this type of mutations can be selected as a first response to the presence of antibiotics. Besides contributing to ceftazidime resistance, the presence of P326Q and P326Q/Q663R also confers resistance to other beta-lactam drugs and do not suppose a fitness cost in an antibiotic-free environment. The SmeH-homolog from *E. coli* AcrB has been a suitable model for predicting the positions of both amino acid residues, showing that P326 is located in the proximities of the deep binding pocket of the efflux pump, while V672, the corresponding Q663 in AcrB, was mapped at the bottom of the access pocket. Taken together, these results suggest that changes in both access and binding pockets might be required for the incremented observed resistance. Whether or not *in vitro* experimental evolution assays are of relevance for understanding evolution during infection in the treated patient is a topic that requires further studies. Nevertheless, a recent analysis of the evolution of carbapenem resistance in *Acinetobacter baumannii* during a prolonged infection in a burn patient shows that a first event in the evolution was the selection of a mutation in a gene encoding a structural component of an efflux pump (*adeB*), followed by the mutation of *ftsI*^82^. This evolution trajectory resembles the one we have observed and supports that the application of experimental evolution and whole-genome sequencing approaches can help to understand and identify the mechanisms of acquired resistance to ceftazidime and other antibiotics by bacterial pathogens.

## Materials and Methods

### Bacterial strains and growth conditions

All the plasmids and strains used in this work are listed in Table 3. The wild-type strain *S. maltophilia* D457 was used as the parental strain for the evolution experiments. Ceftazidime (GlaxoSmithKline) was used at different concentrations during the evolution assay. Cultures were grown using LB medium at 37 °C. Antibiotics were added when required: ampicillin (Ap; 100 µg/ml) for *E. coli* containing the pGEM-T Easy and pGEM-T derived plasmids. Medium was supplemented with 0.5 mM isopropyl-β-D-thiogalactopyranoside (IPTG) and 80 µg/ml 5-bromo-4-chloro-3-indolyl-β-D-galactopyranoside (X-Gal) in order to induce and detect beta-galactosidase production.

**Table 3:** Bacterial strains and plasmids.

Strain or plasmid	Description	Source or reference
**Bacterial strains**		
*E. coli*		
OmniMAX	Strain used in transformation. F’ *mcrA* ∆(*mrr*-*hsd*RMS-*mcr*BC) Φ80(*lac*Z)∆M15 ∆(*lac*ZYA-argF) U169 *end*A1 *sup*E44 *thi*−1 *gyr*A96 *rel*A1 *deo*R *ton*A *pan*D	Invitrogen, Life Technologies
CC118λpir	Donor cell in conjugation. Strain CC118 lisogenized with λ *pir* phage (Tc’) Δ(*ara-leu*), *ara*D, Δ*lac*X74, *gal*E, *gal*K, *pho*A20, *thi*-1, *rps*E, *rpo*B, *arg*E (Am), *rec*A1	^84^
1047 (pRK2013)	Helper cell in conjugation harbouring pRK2013 (Kan^R^) plasmid	^87^
*S. maltophilia*		
D457	Clinical strain	^88^
PBT101	D457 carrying the *smeH* P326Q substitution	This work
PBT102	D457 carrying the *smeH* Q663R substitution	This work
PBT103	D457 carrying the *smeH* P326Q and Q663R substitutions	This work
PBT104	D457 Δ*smeH*	This work
**Plasmids**		
pGEM®-T Easy Vector	Cloning vector, Amp^R^	Promega
pPBT21	pGEM-T-derived plasmid carrying the fragment SmeH:P326R	This work
pPBT22	pGEM-T-derived plasmid carrying the fragment SmeH:Q663R	This work
pEX18Tc	Gene replacement vector; *sacB*, Tet^R^	^83^
pPBT101	pEX18Tc-derived plasmid carrying the fragment SmeH:P326Q	This work
pPBT102	pEX18Tc-derived plasmid carrying the fragment SmeH:Q663R	This work
pHAB5	pEX18Tc-derived plasmid containing the 5′ and 3′ regions of *smeH*	This work

### Experimental evolution

Experimental evolution was performed with the wild-type strain D457 growing in the presence of increasing concentrations of ceftazidime. Cultures were initially grown at the maximum ceftazidime concentration that allowed growth (1 μg/ml) in liquid LB medium. One microliter of a bacterial overnight culture was inoculated in four independent test tubes containing 2 ml of LB medium with ceftazidime Serial passages were performed inoculating 1 μl of bacterial cell cultures in fresh medium containing the same antibiotic concentration every 24 h for 4 days. At day 5, ceftazidime concentration was doubled. This procedure was repeated for 30 days (around 300 generations), until the ceftazidime concentration was 32 μg/ml (32-fold increase). Four independent replicates were also cultured in the same conditions, but in the absence of antibiotic as controls. Every 5 days, and after the passage, the minimum inhibitory concentration (MIC) for ceftazidime was determined for all the evolving populations and samples were taken and kept at −80 °C for further analysis.

### DNA extraction, WGS and identification of mutations

After the 30-days experimental evolution, the total genomic DNAs from the evolved populations and the original wild-type D457 strain were extracted using a Gnome® DNA kit following the manufacturer’s protocol (MP Biomedicals). Quality and quantity of the extracted DNA was assessed by agarose gel electrophoresis and using a NanoDrop Spectrophotometer (Thermo Fischer), respectively. DNA samples were sent to the CRG (Center for Genomic Regulation, Barcelona) and sequenced using the HiSeq2,000 Sequencing System (Illumina) generating 125-bp paired-end reads. The number of reads per sample was 2,093,625 on average, representing a sequencing depth of 100x approximately. Data analysis was accomplished with CLC Genomics Workbench software (Qiagen) and the resulting reads were mapped to the *S. maltophilia* D457 reference genome (NC_017671.1) using default parameters. Single nucleotide polymorphisms (SNPs) detection was performed using the Fixed Ploidy Variant Detection tool (ploidy = 1, required variant probability = 90%, minimum coverage = 8, minimum frequency = 15%) and the given variants were filtered against those obtained for the wild-type D457 strain. Insertions, deletions, inversions, tandem duplications and translocations were detected using the InDel and Structural Variants tool (P-value threshold = 0.0001). The identified mutations were verified by PCR and Sanger-sequencing. Unless otherwise stated, the thermocycler was programmed for 56 °C of annealing. Primers are listed in Supplementary Table S2.

### Recreation of the *smeH* mutations

The amino acid substitutions P326Q and Q663R were introduced alone and in combination in *S. maltophilia* D457. Two 1,000-bp regions of the *smeH* gene, containing each one SNP, were amplified from the ceftazidime evolved population B using primers SmeH_snp1_F and SmeH_snp1_R for Q326 amplification, and SmeH_snp2_F and SmeH_snp2_R for R663 amplification. Both 1,000-bp fragments were cloned into pGEM-T Easy (Promega) following the manufacturer’s instructions obtaining respectively pPBT21 and pPBT22 plasmids, which were introduced by transformation into *E. coli* OmniMAX (Invitrogen). Constructions were verified by DNA sequencing. Both plasmids were extracted with the Qiaprep ® Spin Miniprep Kit 250 (Qiagen) following the manufacturer’s protocol, and digested with EcoRI (New England BioLabs). The obtained products containing either Q326 or R663 were purified and cloned into the suicide vector pEx18Tc^83^, giving rise to pPBT101 and pPBT102, respectively. Both plasmids were then introduced by transformation into CC118*λpir* and selection was performed using tetracycline (4 μg/ml). Then, by tripartite matting, pPBT101 or pPBT102 were introduced into *S. maltophilia* D457^84^ and selection was carried out on LB agar plates containing tetracycline (12 μg/ml) and imipenem (20 µg/ml). Tet^R^ colonies were then streaked onto LB agar plates with 10% sucrose and 12 μg/ml tetracycline. Tet^R^ and Sac^S^ colonies were selected and streaked onto LB plates with 10% sucrose. From these sucrose plates, Sac^S^ colonies were streaked onto LB plates containing sucrose 10% and 12 μg/ml tetracycline with the aim of obtaining the mutants with the amino acid changes. The presence of the mutations was confirmed by SNP-specific PCR; the P326Q change was identified in the PBT101 strain using primers Comp_snp1_F and Comp_snp1_R; the Q663R modification was confirmed in the PBT102 strain using primers Comp_snp2_F and Comp_snp2_R (65 °C annealing temperature). DNA sequencing was performed with primers SmeH_snp1_F and SmeH_snp1_R, and SmeH_snp2_F and SmeH_snp2_R to further confirm the presence of the mutations. Plasmid pPBT102 was introduced as described above in the PBT101 strain in order to obtain a mutant with both amino acid substitutions. Confirmation was carried out in the PBT103 strain with the above-mentioned primers and DNA sequencing. Primers are listed in Supplementary Table S2.

### Generation of a *smeH* deletion mutant

A derivative *S. maltophilia* mutant with a partial deletion in the *smeH* gene was generated through homologous recombination. Using primers HAF and HAR (Table S1), a 494-bp fragment (HA) corresponding to the 5′-end of *smeH* was amplified; primers HBF and HBR were used to amplify a 514-bp fragment (HB) of the *smeH* 3′-end. The thermocycler was programmed for 10 cycles of 52 °C of annealing, followed by 20 cycles of 56 °C. Using HA and HB fragments as templates, an overlapping PCR was carried out using primers HAF and HBR, yielding a 1,000-bp fragment (HAB). The obtained product was cloned into pGEM-T Easy (Promega) and introduced by transformation into *E. coli* CC118*λpir*. DNA sequencing was performed for sequence verification. The plasmid was then digested with EcoRI and the HAB fragment was cloned into pEx18Tc. The resulting plasmid pHAB5 was introduced by transformation into *E. coli* CC118*λpir* and then, by tripartite matting, into *S. maltophilia* D457. The *smeH*-defective mutant was selected following the above-described process and confirmation of the deletion in the PBT104 strain was performed using the primers Ext_smeH_L and Ext_smeH_R, as well as Int_smeH_L and Int_smeH_R. Primers are listed in Supplementary Table S2.

### Antimicrobials susceptibility assay

MICs for the antibiotics cefotaxime, cefoxitin and cefazolin, as well as for menadione, tert-butyl hydroperoxide, hexachlorophene, benzalkonium chloride, triclosan and naringenin, were determined by microbroth double dilution in 96-well microtiter plates (NUNCLON^TM^ Delta Surface) containing LB medium with two-fold dilutions of each antibiotic. Two replicates of each strain were inoculated to a final OD_600_ of 0.01 and plates were incubated for 20 h at 37 °C without shaking. MICs for ceftazidime, aztreonam, ofloxacin, norfloxacin, nalidixic acid, chloramphenicol, tetracycline, tigecycline and polymyxin B were determined using MIC test strips (Liofilchem), which were placed on LB agar plate seeded with an overnight 1:1,000 dilution of each bacterial population. Plates were incubated at 37 °C and results were analyzed after 20 h. The MIC was defined at the lowest concentration at which no bacterial growth was observed.

### Growth curves in the presence of β-lactams

The *S. maltophilia* wild-type strain D457 and the derived mutants PBT101 (SmeH:P326Q), PBT102 (SmeH:Q663R), PBT103 (SmeH:P326Q;Q663R), and PBT104 (Δ*smeH*) were grown in the presence or in the absence of ceftazidime (4 µg/ml), cefoxitin (256 µg/ml) or cefotaxime (128 µg/ml). The assay was carried out in 96-well plates (NUNCLON^TM^ Δ Surface) where 10 µl of an overnight bacterial culture were added to 140 µl of LB medium, with or without the antibiotics, to a final OD_600_ of 0.01. Plates were then incubated at 37 °C for 20 h, and growth (OD_600_) was measured every 10 min using the plate reader Tecan Spark 10 M (Tecan). Shaking for 5 s was performed every 10 min before each measurement.

### Biofilm formation assay

An overnight culture from each tested strain was diluted 1:100 and 100 µl of bacterial suspension were inoculated per well in a 96-well plate (Costar Serocluster^TM^, Corning Incorporated). After 48 h incubation at 37 °C without agitation, biofilms were stained by adding 25 µl of crystal violet 0.1% for 5 min. The stained biofilms were rinsed three times using 100 µl of Milli-Q water and then 150 µl of 0.25% Triton X-100 were added in order to dissociate biofilms. Hundred microliters were transfer to a clean 96-well plate (Nunclon^TM^ Delta Surface) and biofilm formation was assessed through the quantification of crystal violet staining by measuring absorbance at 570 nm. The assay was performed in octuplicate.

### Swimming assay

The swimming motility of all the tested strains was determined on LB agar (0.3%) plates. An overnight culture from each strain was diluted to a final OD_600_ of 2 and 5 µl were spotted on the surface of the swimming plates. After 48 h incubation at 30 °C, the growth zone was measured in milliliters. The assay was performed in triplicate.

### Prediction of the amino acid substitutions location

Structural alignment of SmeH and AcrB amino acid sequences was performed using I-Tasser^85^. the positions of the SmeH amino acid substitutions were represented using the AcrB crystal structures of *E. coli* as a reference (PDB ID codes 4DX7.A and 4DX7.B^37^). Prediction of both access and deep binding pocket cavities in AcrB was performed with CASTp^86^. Figures were generated using PyMol (The PyMOL Molecular Graphics System, Schrödinger, LLC).

## Supplementary information

Supplementary information
