# Supplementary material for: Involvement of the RND efflux pump transporter SmeH in the acquisition of resistance to ceftazidime in Stenotrophomonas maltophilia
Source: Sci Rep. 2019 Mar 20;9:4917. doi: 10.1038/s41598-019-41308-9 (PMC6426872; doi:10.1038/s41598-019-41308-9)
Supplement: Supplementary file 1 — Supplementary information [file 41598_2019_41308_MOESM1_ESM.pdf]

Involvement of the RND efflux pump transporter SmeH in the acquisition of resistance to ceftazidime in *Stenotrophomonas maltophilia*

Paula Blanco, Fernando Corona, José Luis Martínez

Supplementary information.

Table S1. MICs of several antibiotics for *S. maltophilia* strains

Strain	MIC (μg/ml)											
	**CAZ**	**CTX**	**FOX**	**CFZ**	**ATM**	**OFX**	**NOR**	**NAL**	**CHL**	**TET**	**TGC**	**PMB**
D457	0.75	128	256	4,096	4	1	8	6	6	1.5	0.5	8
PBT101 (P326Q)	4	192	384	8,192	8	1	8	6	8	1.5	0.75	8
PBT102 (Q663R)	0.75	96	256	3,072	6	1	8	6	6	6	0.75	12
PBT103 (P326Q; Q663R)	8	256	384	8,192	24	1	24	6	4	2	0.5	8
PBT104 (*ΔsmeH*)	0.38	96	256	3,072	2	0.5	6	4	6	1	0.5	3

MIC, minimum inhibitory concentration; CAZ, ceftazidime; CTX, cefotaxime; FOX, cefoxitin; CFZ, cefazolin; ATM, aztreonam; OFX, ofloxacin; NOR, norfloxacin; NAL, nalidixic acid; CHL, chloramphenicol; TET, tetracycline; TGC, tigecycline; PMB, polymyxin B.

Table S2. Primers used in this study

Name	Sequence (5'-3')	Utilization
SmeH_snp1_F	GAATTCAGTACGCCATGAACATCTGG	Amplification of the 1000-bp fragment containing P326Q in *smeH*
SmeH_snp1_R	GAATTCACTTGTTGAAGGTGCGGTAG	
SmeH_snp2_F	GAATTCCCGGTGAGATCTACAAGCAG	Amplification of the 1000-bp fragment containing Q663R in *smeH*
SmeH_snp2_R	GAATTCGACTTGACCACCGTGTTGAG	
Comp_snp1_F	GTGACCTGGTTCTCGCA	Verification of Q326
Comp_snp1_R	GAACAGGGTCAGCTGGTT	
Comp_snp2_F	ACCTGCCCACCGTGCG	Verification of R663
Comp_snp2_R	CGGCCTTCGTAGAAGAAGTCGTT	
HAF	GAATTCCGAACATCCAGTCTTCGCCTG GGTGGTTGCGATCCTG	Amplification of the 489-bp fragment corresponding to the 5'-end of *smeH*
HAR	AGCTTTCATACAGGGCGGCCGGGGAT ACGCGAGACCTGGT	
HBF	ACCAGGTCTCGCGTATCCCCGGCCGCC CTGTATGAAAGCT	Amplification of the 489-bp fragment corresponding to the 3'-end of *smeH*
HBR	GAATTCTCAACGATCCGGGCGGTGTG CAGCATCCGC	
Ext_smeH_L	GATTCGAACAAGCAGTAAG	Amplification of the 3404-bp fragment including the complete *smeH* gene
Ext_smeH_R	ATTGGTAATCGTGGCAGTGT	
Int_smeH_L	ACAACTACGGCTTCGACAC	Amplification of a 211-bp fragment inside *smeH* gene
Int_smeH_R	GTCTTGACCACTTCCTGGAT	
acrD1_L	GTCGGCCAGCCAGGTACT	Verification of P326Q in *smeH*
acrD1_R	ATCTGGGTCATCGCCTTCT	

acrD2_L<br>acrD2_R	ATGATCCTGTTCGTGGTGCT<br>GAAGCTCTTCAGCGACTCCT	Verification of Q663R in *smeH*
phoQ_cef_L<br>phoQ_cef_R	ACCCTGGCGATAACACGAT<br>ACCTTGGTCAACTCGGTGAT	Verification of I76N and P81L in *phoQ*
phoQ_tig3_L<br>phoQ_tig3_R	CGAATTCCCGTACACCATCT<br>CACTTGAAGGCGTTCTCCAG	Verification of S307L and P324L in *phoQ*
mrkC_L<br>mrkC_R	GCTGGCACCTACCGTGTC<br>AGATCAGCTGCTGGTTCTGG	Verification of G187_F188del in *mrkC*
ftsI4_L<br>ftsI4_R	CAGATCATCGACGAGAACG<br>AACATCGAAGGACTCATTGC	Verification of A592D in *ftsI*
yrbE_L<br>yrbE_R	GTTCTCGCTGACCGTGCT<br>GTGAACAACATTGCCGACAG	Verification of I49fs in *yrbE*
yrbC_L<br>yrbC_R	GTCGGCAAGTACATGTTCAG<br>AAGTGCGTTACTTGCCATTG	Verification of Q126* in *yrbC*
yciM_L<br>yciM_R	ATCGAAGATGGATTTCGTCA<br>AGGTCAGCAACGCACTGA	Verification of A206fs in *yciM*
SMD_0534_L<br>SMD_0534_tig_R	TGCGGAATTTTTCGAGTATC<br>AGGTTGGAGATGGACTGTGG	Verification of V398fs in *smd_0534*
SMD_1278_L<br>SMD_1278_R	CCCAACCACTCTTCTGTGG<br>CGTTCCTGGTACTCCTTGAC	Verification of F91_G92insDF in *smd_1278*
SMD_0260_L<br>SMD_0260_R	GATTGGAACCTGGTCGAG<br>TCTTCGACCAGAAGGATACG	Verification of K88R in *smd_0260*

SMD_2719_L	GCAACGGGATCTGGGT	Verification of V232G in *smd_2719*
SMD_2719_R	ACCACGAACGGCAGCTTG	

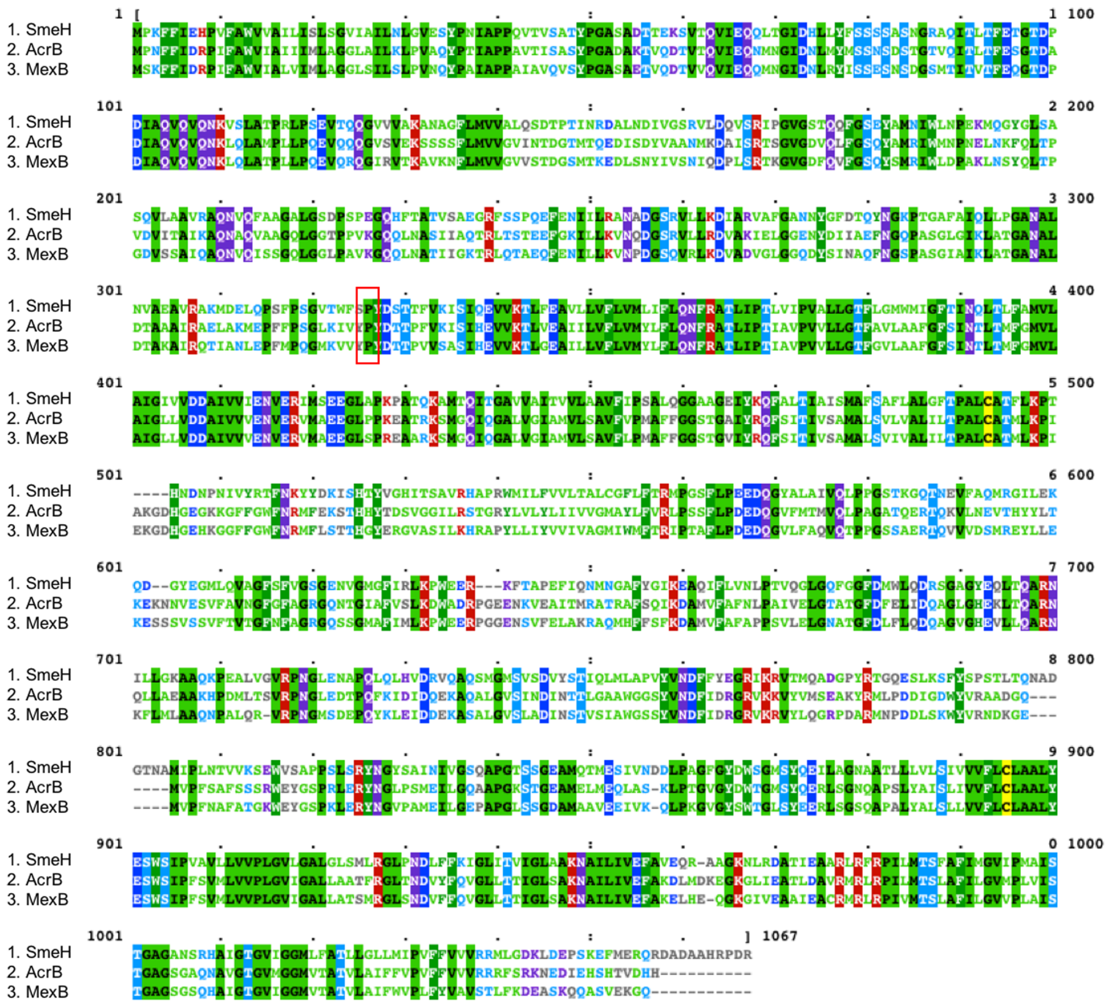

Figure S1. Alignment of the *S. maltophilia* SmeH amino acid sequence with its homologues AcrB from *E. coli*, and MexB from *P. aeruginosa*. The conserved P326 residue is marked with a red square in the amino acid sequences of the three species. Color scheme is based on amino acid identity.
